# Harnessing human immune system models to validate NADPH oxidase 1 inhibition as treatment for hepatocellular carcinoma

**DOI:** 10.3389/fphar.2026.1808515

**Published:** 2026-07-14

**Authors:** Zenzi De Vos, Lander Heyerick, Aline Baekelandt, Lara Morren, Anne Hoorens, Hasan Eker, Luís Abreu de Carvalho, Filip Gryspeerdt, Frederik Berrevoet, Hannah Stocks, Andy Wullaert, Sarah Raevens, Anja Geerts, Xavier Verhelst, Sander Lefere, Hans Van Vlierberghe, Lindsey Devisscher

**Affiliations:** 1 Gut-Liver Immunopharmacology Unit, Department of Basic and Applied Medical Sciences, Ghent, University, Ghent, Belgium; 2 Liver Research Center Ghent, Ghent University, Ghent University Hospital, Ghent, Belgium; 3 Cancer Research Institute Ghent, Ghent, Belgium; 4 Department of Pathology, Ghent University Hospital, Ghent, Belgium; 5 Department of General and HPB Surgery and Liver Transplantation, Ghent University Hospital, Ghent, Belgium; 6 Cell Death Signaling Laboratory, Department of Biomedical Sciences, University of Antwerp, Antwerp, Belgium; 7 VIB-UGent Center for Inflammation Research, VIB, Ghent, Belgium; 8 Department of Gastroenterology and Hepatology, Ghent University Hospital, Ghent, Belgium; 9 Hepatology Research Unit, Department of Internal Medicine and Paediatrics, Ghent University, Ghent, Belgium

**Keywords:** adaptive immunity, cancer immunity, hepatocellular carcinoma, human immune system mouse models, humanized models, innate immunity, NADPH oxidase 1, precision-cut tumor slices

## Abstract

**Introduction:**

NADPH oxidase 1 inhibition (NOX1i) has shown to alter the tumor microenvironment in conventional mouse models for hepatocellular carcinoma (HCC). However, clinical translation is hampered by low translatability of these models.

**Methods:**

We developed two novel human immune system (HIS) mouse models, co-transplanted with orthotopic human HCC cells, to investigate NOX1i in the context of human HCC and human adaptive (T cell-HIS-HCC) or innate (Myeloid-HIS-HCC) immune responses. Mice received NOX1i or vehicle twice per week for 3 weeks. Results were validated in *ex vivo* patient-derived precision-cut tumor slices (PCTS).

**Results:**

T cell-HIS-HCC mice were mainly reconstituted with human T cells. Interestingly, the expression of cytokines and markers involved in both cancer progression and anti-tumor immunity was significantly lower in tumors of NOX1i-treated mice. In the Myeloid-HIS-HCC model, humanization in livers and tumors was dominated by macrophages. Significantly lower human immune cells were observed in tumors of NOX1i-treated Myeloid-HIS-HCC mice, in line with reduced *VCAM1* and *ICAM1* expression. NOX1i-treated Myeloid-HIS-HCC mice showed a similar shift in tumor-promoting cytokines and immune checkpoints as NOX1i-treated T cell-HIS-HCC mice. Moreover, gene expression of tumor-associated macrophage, HCC and proliferation markers tended to be lower in tumors of NOX1i-treated Myeloid-HIS-HCC mice*. Ex vivo* patient-derived PCTS treated with NOX1i also demonstrated lower gene expression of pro-tumorigenic cytokines and tumor-promoting markers.

**Conclusion:**

Our data show that NOX1i modulates the tumor-immune microenvironment in human immune system HCC models, and might hold potential in combination therapy to rebalance the dysregulated immune profile in HCC.

## Graphical Abstract

**Figure:**
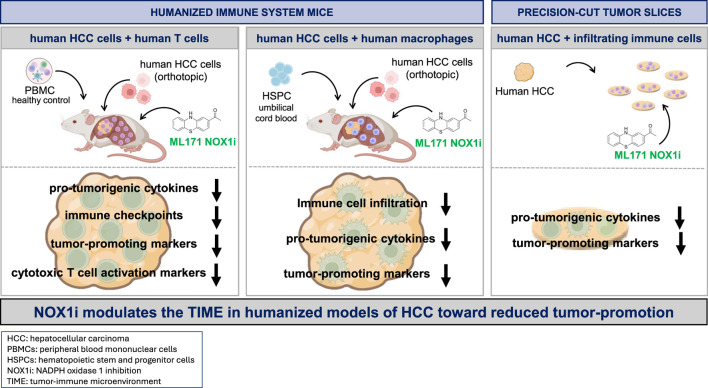

## 1 Introduction

Hepatocellular carcinoma (HCC) is the most prevalent type of primary liver cancer and typically develops on a background of chronic liver inflammation (Singal et al., 2023; Llovet et al., 2016). Under normal physiological conditions, immunity in the liver is marked by tolerance owing to sustained exposure to harmless gut-derived antigens. The tolerogenic nature of the liver and chronic inflammation in the vast majority of HCC patients collectively result in the development of a unique tumor-immune microenvironment (TIME) characterized by the presence of suppressive immune cells (Donne and Lujambio, 2023; Di Giacomo et al., 2025). The suppressive TIME contributes to relapse and the lack of effective treatment strategies, emphasizing the need for new treatment options for HCC patients (Ringelhan et al., 2018). Given the suppressive TIME in HCC, targeting maladaptive immune responses or cells associated with the dysregulated immune profile pose interesting targets for HCC therapy.

Chronic inflammation, immunosuppression and the hypoxic environment in HCC are associated with reactive oxygen species (ROS) imbalance. While low and balanced ROS levels act as secondary messenger and are essential for normal cell function, including effective immune responses, increased ROS levels, i.e., during chronic inflammation, impair both innate and adaptive immune responses and create a tumor-permissive environment (Shah et al., 2024; Begum et al., 2022; Panday et al., 2015; Glorieux et al., 2024; Cheung and Vousden, 2022). The rapid growth and metabolism of tumor cells increase ROS levels and induce oxidative stress. This further supports the dysregulated immune response, thereby compromising anti-tumor immunity and promoting cancer development and growth (Roy et al., 2015; Begum et al., 2022; Panday et al., 2015; Cheung and Vousden, 2022; Shah et al., 2024). During homeostasis, ROS levels are strictly regulated by antioxidant and ROS-producing mechanisms. NADPH oxidase (NOX) enzyme complexes are major producers of non-mitochondrial-derived ROS in response to inflammatory cytokines, i.e., during (chronic) inflammation (Roy et al., 2015). Thus, inhibition of NOX enzymes poses a promising therapeutic strategy for HCC (Panday et al., 2015). Of all 7 NOX isoforms (NOX1-5 and DUOX1-2) (Roy et al., 2015), NOX1 has been associated with poor prognosis in HCC patients (Ha et al., 2016; Eun et al., 2017). Interestingly, macrophage differentiation and polarization is affected in NOX1/NOX2 double knock-out mice (Xu et al., 2016), and genetic depletion of NOX1 in macrophages in a murine HCC model has shown to protect the liver from HCC development (Liang et al., 2019), together suggesting a role of NOX1 in modulating the immunosuppressive TIME in HCC. Indeed, our research group and Liang et al. (Vandierendonck et al., 2021; Liang et al., 2019) have shown that pharmacological inhibition of NOX1 reduces the pro-inflammatory, angiogenic and pro-fibrotic tumor microenvironment (TME) of HCC and attenuates tumor growth in conventional murine HCC models.

Despite substantial overlap between human and murine immunity, differences in immune cell subsets, cytokine production, receptor expression, cell signaling and genomic expression indicate that murine immunity is not representative of human immunity, and that conventional HCC models fail to accurately recapitulate the dysregulated immune responses that characterize the TIME of human HCC (Tao and Reese, 2017; Mestas and Hughes, 2004; Gros and Casanova, 2023; Courau et al., 2026). While 3D models, including spheroids and organoids, are gradually replacing 2D models in various research fields, co-culture of 3D systems with immune cells is challenging and inadequately reflects the TIME (Mu et al., 2023). Nevertheless, *ex vivo* precision-cut tissue slices retain the *in vivo* histoarchitectural structure, including immune cells and cell-cell and cell-matrix interactions (Olinga and Schuppan, 2013; De Graaf et al., 2010; Davies et al., 2015; Jagatia et al., 2023), and are a promising tool to study tumor-immune interactions. Furthermore, humanized immune system (HIS) mouse models allow *in vivo* investigation of human immune cells, and have been utilized to study various diseases, including cancer (Chuprin et al., 2023). HIS mice are immunodeficient mice in which part of the immune system is reconstituted with human immune cells. The most thoroughly investigated HIS model employs human hematopoietic stem and progenitor cells (HSPCs), which results in high engraftment of T cells, accompanied by minimal engraftment of innate immune cells. Notably, T cells in human HSPC-reconstituted HIS mice are educated in a murine thymus, which impairs their functionality. Alternatively, human T cell engraftment is achieved through humanization with human peripheral blood mononuclear cells (PBMCs). While PBMC-humanized mice engraft mature T cells educated in a human thymus, their applicability is hampered by rapid onset of graft-versus-host disease (Chuprin et al., 2023). Overall, the use of HIS models in HCC is limited and mainly reports human T cell engraftment from HSPCs (Weinfurtner et al., 2025; Zhao et al., 2021).

Considering that current HIS mouse models fail to reconstitute physiologically relevant levels of both adaptive and innate immune cells in one model, and the limited use of such models including substantial numbers of innate immune cells, we developed two separate, complementary HCC models that engraft mature T cells (adaptive arm) or tumor-associated macrophages (TAMs; innate arm), representing crucial human immune cell subsets in HCC (Wang et al., 2023; Zheng et al., 2017).

In this study, we provide evidence for NOX1i as treatment strategy for human HCC by leveraging these two HIS mouse models that interrogate human HCC in the context of human adaptive or innate immunity. To increase clinical translation, NOX1i was additionally validated in *ex vivo* patient-derived precision-cut tumor slices (PCTS).

## 2 Materials and methods

### 2.1 Human samples

Human healthy control buffy coat was obtained from the Red Cross Flanders (Ghent, Belgium). Human umbilical cord blood (CB) was obtained from the CB bank of Ghent University Hospital (UZ Ghent). Treatment-naïve HCC patients (n = 3) undergoing resection were selected at UZ Ghent (Supplementary Table S1), and viable HCC tissue from the resected residual body material was collected at the Department of Pathology (UZ Ghent) after written informed consent in compliance with the Declaration of Helsinki. The use of human immune cells and human HCC tissue was reviewed and approved by the Medical Ethics Committee of Ghent University and UZ Ghent (approval BC-11115 and BC-2015/1334).

### 2.2 Human immune cell isolation

Buffy coat was diluted 1/5 with Dulbecco’s phosphate buffered saline (DPBS, Gibco, Fisher Scientific S.R.L., #14200075). Peripheral blood mononuclear cells (PBMCs) were isolated from diluted buffy coat based on density gradient using Lymphoprep (STEMCELL Technologies, #07811). Isolated PBMCs were washed twice with DPBS, and cells were counted manually using a Bürker chamber (Novolab, #A14828). PBMCs were frozen in Roswell Park Memorial Institute (RPMI) 1640 supplemented with GlutaMAX (Gibco, #61870010), 10% Heat Inactivated Fetal Bovine Serum (FBS; Gibco, #A5669801) and 10% dimethyl sulfoxide (DMSO; Sigma-Aldrich, Merck Life Science BV, #D2438), at a density of 30–60 × 10^6^ cells per cryovial (Greiner Bio-One, #122263). Cryovials were transferred to −80 °C in a CoolCell (Corning, #432001) to allow controlled-rate freezing of −1 °C/min, and vials were transferred to a liquid nitrogen tank after at least 24h for long-term storage.

Freshly isolated CB-derived PBMCs, isolated from CB as described for buffy coat, were enriched for HSPCs based on positive immunomagnetic selection of CD34^+^ expressing cells using the human CD34 MicroBead Kit UltraPure (Miltenyi Biotec, #130–100-453) according to manufacturer’s guidelines. To increase the purity of HSPC enrichment, cells were loaded on the column in the magnetic field twice. Injected HSPC fractions contained at least 98% CD34^+^ cells and less than 0.05% T cells, as determined by flow cytometry (Supplementary Figure S1). CB-derived HSPCs were frozen in 90% FBS supplemented with 10% DMSO, in a CoolCell at −80 °C. Frozen cells were thawed in pre-warmed RPMI 1640 supplemented with Glutamax and 10% FBS, and washed twice with DPBS before use.

### 2.3 Cell culture

The human male HCC cell line Hep3B (Cytion, #305141) was maintained in Dulbecco’s Modified Eagle Medium (DMEM) with GlutaMAX, 4.5 g/L D-Glucose and sodium pyruvate (Gibco, #31966047), supplemented with 10% FBS and 1% antibiotic/antimycotic (A/A; Gibco, #15240062). When reaching 75%–80% confluency, cells were split using Trypsin-EDTA (0.05%; Gibco, #25300062). Cells were maintained at 37 °C in a humidified atmosphere with 5% CO_2_. Cell stocks were frozen in DMEM supplemented with 50% FBS and 10% DMSO, in a coolcell at −80 °C, and stored long-term in liquid nitrogen. Hep3B cells were thawed at least 1 week prior to tumor induction, and were used at passage 24–25. Cells tested negative for *mycoplasma* contamination (Core Clinical Tissue and Cell Culture at Ghent University, Belgium).

### 2.4 Mice

Immunodeficient NOD-scid IL-2Rgamma^null^ (NSG, JAX stock #005557) mice and NSG mice with transgenic and homozygous expression of human IL-3, human GM-CSF and human SCF (NSG-SGM3, JAX stock #013062) were bred by the animal facility and PDX Core facility of the Faculty of Medicine and Health Sciences of Ghent University. Only male offspring were used for experiments. A total number of 23 male mice were used in this study. Mice were housed in individually ventilated cages (IVC) in a temperature-controlled room with 12 h light/dark cycle at the animal facility of the Faculty of Medicine and Health Sciences (Ghent University). Mice had *ad libitum* access to mouse maintenance chow (irradiated, Bio-Services B.V. #V1124-703) and water, and received care in accordance with the ‘Guide for the Care and Use of Laboratory Animals’ and the Belgian national guidelines for animal protection. All experiments were reviewed and approved by the Animal Ethics Committee of Ghent University, faculty Medicine and Health Sciences (approved ECD 21/61 and ECD 24/09) and are reported according to ARRIVE guidelines (2.0).

### 2.5 T cell-HIS-HCC mouse model

Orthotopic human HCC was induced in nine-week-old male NSG mice (n = 12) via slow intrahepatic injection of 1.3 × 10^6^ Hep3B cells suspended in 30 µL 1:1 DPBS/Matrigel (Basement Membrane Matrix, High concentration; Corning, #354248), under isoflurane anesthesia (5% v/v for induction, 2% v/v for maintenance, oxygen as carrier gas). Two weeks post tumor induction, mice were humanized via IP injection of 8 × 10^6^ human buffy coat-derived PBMCs suspended in 150 µL DPBS. Four days post-humanization, mice were treated with 50 µM ML171 (selective NOX1 inhibitor; Medchem Express, #HY-12805) or vehicle (2% DMSO) in 100 µL DPBS per mouse, via intraperitoneal injection, twice a week for 3 weeks. Mice were randomized to treatment (NOX1i, n = 6) or control groups (n = 6) using RandoMice software. Researchers were blinded for treatment identity at administration and sample processing. Mice were euthanized 4 weeks post tumor induction. No animals met the exclusion criteria of failed tumor induction. Four mice showed failed human engraftment, predefined as below 25% in tissue, and were excluded from all analyses (Supplementary Figures S2A, S2B).

### 2.6 Myeloid-HIS-HCC mouse model

Human myeloid development in immunodeficient mice is achieved via transgenic expression of three human myelopoiesis-promoting genes (NSG-SGM3 mice) (Wunderlich et al., 2018), enabling evaluation of *in vivo* innate immune responses in HCC. Myeloid cells derive from HSPCs in this model, which is accompanied by slower engraftment kinetics compared to engraftment using mature T cells in the T cell-HIS model. Therefore, human immune cell reconstitution was performed prior to tumor induction to allow optimal myeloid engraftment during the treatment period. To this end, six-week-old male NSG-SGM3 (n = 11) mice were sublethally irradiated with 100 cGray (Precision SmART + Irradiator, SmART Scientific Solutions). After 6 hours, 7 × 10^5^ thawed human CB-derived HSPCs, suspended in 100 µL DPBS, were injected intravenously. After 4 weeks, 20–40 µL peripheral blood was collected in K3 EDTA tubes (Microvette, Sarstedt, #20.1278.100) from the lateral tail vein to evaluate humanization. Mice were included for further experiments if humanization exceeded 7.5% human CD45^+^ cells in peripheral blood (pre-defined criterium, Supplementary Figures S2A, S2C). Orthotopic human HCC tumors were induced and mice were treated with NOX1i or vehicle as described for T cell-HIS-HCC mice. Mice were allocated to the treatment or control group based on humanization level at week four using RandoMice software. Mice were euthanized 4 weeks post tumor induction. No animals met the exclusion criteria of failed tumor induction or absent human engraftment, predefined as below 7.5% in blood (Supplementary Figures S2A, S2D). Therefore, all animals were included in the analyses.

### 2.7 Tissue sampling

Peripheral blood was collected in K2E EDTA tubes (Microtainer, BD, #365974) for flow cytometric analysis. Orthotopic liver tumors were measured in two dimensions (length and width) and tumor volume was determined according to the formula ‘volume = ½ (length x width^2^)’. Liver and tumor tissue for flow cytometric analysis was flushed with ice-cold DPBS and stored in ice-cold RPMI 1640 with L-Glutamine (Gibco, #11875–119) until further processing. Tissue for histology was stored in 4% phosphate-buffered formaldehyde solution (VWR, Avantor) for 24h, and further in DPBS until processing. Tissue for gene expression analysis was stored in RNAlater solution (Invitrogen, Fisher Scientific S.R.L., #AM7021), snap frozen and stored at −80 °C until further processing.

### 2.8 Immunophenotyping by flow cytometry

Red blood cells were removed from peripheral blood through incubation with 1 mL red blood cell (RBC) lysis buffer for 10 min at room temperature (RT). Single cell suspensions from liver and tumor tissue were obtained after enzymatic digestion with 1 mg/mL Collagenase A (Sigma-Aldrich, #10103586001) and 300 μg/mL DNAse I (Roche Diagnostics, #10104159001) dissolved in 3 mL RPMI with L-Glutamine, and mechanical digestion using GentleMACS C Tubes and dissociator (Miltenyi Biotec) according to manufacturer’s instructions. Cells were passed through a 100 µm filter, and incubated with 2 mL RBC lysis buffer for 45 s. Cell clumps were removed using a 40 µm filter, and single cell suspensions were plated in a 96-well plate (V-bottom) and stained with 1/800 Zombie Aqua (fixable viability dye) for 15 min at RT. Fc receptors (FcR) were blocked with 1/100 murine and human anti-FcR block to prevent unspecific binding of fluorochrome-labelled antibodies. Intracellular proteins FOXP3 and CD68 were stained using eBioscience™ Foxp3/Transcription Factor Staining Buffer Set (Invitrogen, #50–112-8857) according to manufacturer’s instructions. Samples were subjected to one or more panels: Hematopoietic stem and progenitor cell purity panel, human immune cell chimerism panel, human monocyte/macrophage panel or human T cell-specific panel. The antibodies used for each panel are listed in Supplementary Table S2, and representative gating strategies are shown in Supplementary Figures S1A, S2A, S3B, S6. Counting Beads were used to determine cell numbers and were added 1/10 for blood and 1/15 for tissue. All antibodies, FcR blocks (mouse #156604, human #422302), live/dead stain (#423102) and counting beads (#424902) were purchased at Biolegend. Samples were measured on the BD FACSSymphony A3 flow cytometer (BD Benelux) and analyzed using Flow Jo software (v10.8.1).

### 2.9 Histology

Buffered formaldehyde-fixed tissue was processed using an automated tissue processor (HistoCore PEARL, Leica Biosystems) prior to embedding in paraffin. Paraffin-embedded blocks were sliced in 4 µm sections using the RM2145 microtome (Leica Biosystems), and were used for hematoxylin and eosin staining or immunohistochemistry staining of human CD14.

#### 2.9.1 Hematoxylin and eosin staining

Sections were stained with hematoxylin (Sigma-Aldrich, #MHS128) and eosin (Sigma-Aldrich, #E4382) using an automated staining program on the ST5020 and CV5030 multistainer (Leica Biosystems).

#### 2.9.2 Immunohistochemistry staining

Immunohistochemistry was performed on deparaffinized and rehydrated sections after antigen retrieval and blocking of endogenic peroxidase, using a monoclonal primary antibody detecting human CD14 (Cell Signaling Technologies (BIOKé), #D7A2T) at 10 ng/mL. Primary antibody was detected via SignalStain® Boost IHC Detection Reagent (HRP, Rabbit; Cell Signaling Technologies, #8114) and SignalStain® DAB Substrate Kit (Cell Signaling Technologies, #8059) according to manufacturer’s instructions. Nuclei were counterstained with hematoxylin (Sigma-Aldrich, #MHS128). As negative control, serial sections were processed via identical protocol using Rabbit mAb IgG XP® Isotype Control (Cell Signaling Technologies, #DA1E) as primary antibody.

### 2.10 Immunofluorescence staining

Snap-frozen tumor tissue from humanized mice was fixed in AntigenFix (DiaPath, #P0016) for 1 h at 4 °C, washed twice in DPBS, incubated overnight in 34% sucrose (Sigma-Aldrich, #S9378) and frozen in OCT Mounting media (VWR, Avantor, #361603E). Sections (15 µm) were cut using a cryostat (CM 1950, Leica Biosystems) and rehydrated in DPBS for 5 min. Tumor sections were permeabilized and blocked for 30 min with blocking buffer 1 containing 0.5% saponin (Sigma-Aldrich, #47036), 2% bovine serum albumin (BSA; #A9418), 1% FBS and 1% normal goat serum (Abcam, #ab7481), prior to overnight incubation with primary rabbit anti-human CD68 (1/100 dilution in blocking buffer; Abcam, #ab213363) or rabbit anti-human CD3 zeta (1/250 dilution in blocking buffer; Abcam, #ab68235) antibody and was visualized using secondary goat anti-rabbit IgG Fc-Alexa fluor 594 (1/300 dilution in blocking buffer; Abcam, #ab150092) antibody. Sections were subsequently permeabilized and blocked in blocking buffer 2 containing 0.5% saponin, 2% BSA, 1% FBS and 1% normal rabbit serum (Abcam, #ab166640), and incubated with conjugated rabbit anti-human CD163-Alexa Fluor 488 (1/100 dilution in blocking buffer 2, Abcam, #ab218293) or rabbit anti-human Granzyme B-Alexa Fluor 488 (1/100 dilution in blocking buffer 2, Abcam, #ab225472) antibody. Nuclei were stained with DAPI (0.2 μg/mL in DPBS; Sigma-Aldrich, #D8417). Sections were mounted in Fluoromount (Sigma-Aldrich, #F4680) and imaged using a fluorescence microscope (Eclipse Ni, Nikon). Images were obtained with Fiji software (ImageJ, v.2.9.0).

### 2.11 Reverse transcription qPCR

Total RNA was extracted from tumor tissue using TissueLyser LT (Qiagen #85600) and Aurum Total RNA Mini Kit (Bio-Rad, #7326820), according to manufacturer’s guidelines. Quality and concentration of the isolated RNA was measured on a spectrophotometer (NanoDrop 1000, Thermo Scientific). RNA was used to synthesize complementary DNA (cDNA) with the Bioline SensiFast cDNA Synthesis Kit (GC Biotech, #BIO-65054). Gene expression of the indicated cytokines, inflammatory and tumor markers was analyzed in duplicate, using human primers listed in Supplementary Table S3 and the Bioline Sensimix Sybr No-ROX kit (GC Biotech, #QT650-20), according to manufacturer’s instructions. Primers were screened for human-specific amplification using primer-BLAST (Ye et al., 2012). Cq values were measured with the Lightcycler 480 II (Roche Diagnostics). Fold change was calculated using the ΔΔCq method, and gene expression was normalized to the expression of *HPRT, HMBS, SDHA* and *ACTB*. Where indicated, fold changes were additionally normalized to the number of engrafted human immune cells in the tumor of the respective mouse.

### 2.12 Precision-cut tumor slices

HCC tissue from three HCC patients (patient characteristics are listed in Supplementary Table S1) was obtained at the Department of Pathology (UZ Ghent), and stored on ice in Krebs Henseleit buffer (NaCl (118 mM; Merck #1064040500), KCl (5 mM, Merck, #1049361000), MgSO_4_.7H_2_O (1.1 mM, Merck, #1058861000), KH_2_PO_4_ (1.2 mM, Merck #1048731000), CaCl_2_.2H_2_O (2. mM, Sigma-Aldrich, #C7902-500G), D-Glucose monohydrate (25 mM, Merck, #1083421000), NaHCO_3_ (25 mM, Merck, #1063291000) and HEPES (9 mM, Gibco, #15630–056) in sterile water) until processing. Slices of 250 µm thin were obtained using a vibratome (VT1200S, Leica Biosystems) at an angle of 18°, amplitude of 3 mm and speed of 0.1 mm/s. Unsliced tumor tissue was kept in oxygenized Krebs Henseleit buffer until slicing, and tumor tissue and slices were kept ice-cold during the whole slicing procedure. Uniform slices of 5 mm in diameter were cut using a biopsy punch (Stiefel, Fisher Scientific, #10219839). Precision-cut slices were transferred to oxygenized ice-cold William’s E Medium with GlutaMAX (Gibco, #32551020) supplemented with 5% human AB serum (Sigma, #H4522), 1x ITS-G (insulin, transferrin, selenium; Gibco, #41400045), 0.36 μg/mL Glucagon (Sigma-Aldrich, #G2044), 6.2 ng/mL human recombinant EGF (STEMCELL Technologies, #78006.1), 1x A/A and 50 μg/mL Gentamycin (B.Braun, #3521920; WME complete (WMEc)).

PCTS (n = 22) from three HCC patients were cultured in WMEc on an orbital shaker at 90 rpm, in a humidified incubator at 37 °C and 5% CO_2_. Each slice was cultured in a 24 well containing 900 mL WMEc. PCTS were precultured for 2 h and transferred to fresh medium containing either 5 µM ML171 (NOX1i; n = 11) or vehicle (DMSO, n = 11) for 6 h. PCTS were collected in RNAlater, snapfrozen in liquid nitrogen and stored at −80 °C.

### 2.13 Reverse transcription qPCR of precision-cut tumor slices

Total RNA was extracted from PCTS using the RNeasy Micro kit (Qiagen) according to manufacturers’ instructions following mechanical tissue disruption utilizing a TissueLyser LT (Qiagen #85600). Quality and concentration of RNA were checked, cDNA was prepared and gene expression was analyzed as for tumor tissue, as listed above.

### 2.14 Statistics

All statistical tests were carried out in GraphPad Prism (v10.4.0). Normal distribution of the data was evaluated with Shapiro-Wilk and Kolmogorov-Smirnov tests. P-values of normally distributed data comparing two groups were calculated using an unpaired parametric t-test with Welch’s correction, and for not normally distributed data, the non-parametric Mann-Whitney U test was used. For pairwise comparison of several groups, adjusted p-values were calculated using multiple unpaired t-test with Holm-Šídák correction. Where indicated and in cases of normality, F-test was used to compare variances. Used statistical tests are indicated in figure legends. Donor variability in PCTS was assessed using one-way ANOVA per gene per condition. (Adjusted) p-values were considered statistically significant if below 0.05. Sample size calculations were carried out with G*Power (v3.1).

## 3 Results

### 3.1 NOX1i alters the tumorigenic environment in a T cell-his-HCC mouse model

**FIGURE 1:**
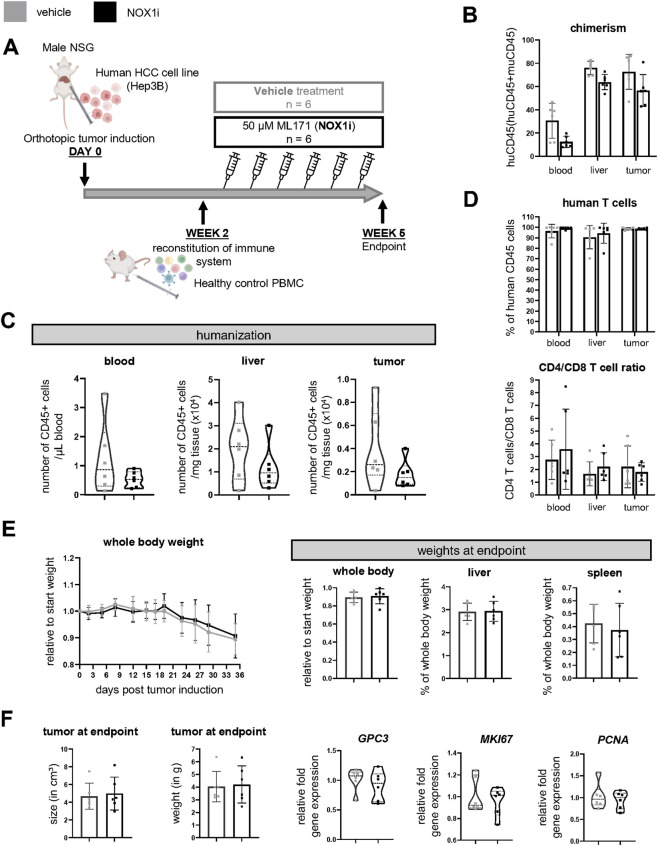
NOX1i does not affect tumor growth in T cell-HIS-HCC mice. **(A)** Schematic overview of the experiment. Orthotopic human HCC tumors were induced in male NSG mice (n = 12), and the immune system was partly reconstituted 2 weeks later using human healthy control PBMCs. Mice were treated with 50 µM NOX1i (n = 6) or vehicle (n = 6), twice per week, for 3 weeks. **(B)** Human-to-murine immune cell (CD45) chimerism in the indicated tissues. **(C)** Human engraftment based on the number of human CD45 cells. Data are represented as violin plots showing median and quartiles. **(D)** Human engraftment based on percentage of T cells from human CD45, and human CD4-to-CD8 T cell ratio. **(E)** Whole body weight relative to start weight during the experiment and at endpoint, and liver and spleen weight represented as percentage of whole body weight at endpoint. **(F)** Tumor growth based on tumor weight and size, and gene expression of *glypican 3* (*GPC3*), *Ki67* (*MKI67*) and *PCNA*. **(B,D–F)** Data are shown as mean (SD). **(C–F)** Adjusted p-values were calculated using multiple unpaired t-test with Holm-Šídák correction **(D,E)**, or unpaired t-test with Welch’s correction **(C,F)** or unpaired Mann-Whitney U test **(F)** based on normal distribution. HCC: hepatocellular carcinoma, PBMC: peripheral blood mononuclear cells, NOX1i: NOX1 inhibition, huCD45: human CD45, muCD45: murine CD45.

Previous literature states a role for NOX1 in monocyte and macrophage differentiation and function (Xu et al., 2016; Liang et al., 2019), while data and evidence on murine and particularly human T cells is lacking. To investigate whether NOX1i affects mature T cells in the context of human HCC, we developed a T cell-HIS-HCC mouse model. For this, orthotopic human HCC was induced in NSG mice, and humanization was achieved using PBMCs. Mice were treated with NOX1i or vehicle, twice a week for 3 weeks (Figure 1A).

Humanization was successful, and showed mean human-to-murine immune cell chimerism of 30.6%, 75.9% and 72.6% in peripheral blood, liver and tumor, respectively, in vehicle-treated mice (Figure 1B). Notably, NOX1i tended to reduce human immune cell engraftment in peripheral blood, liver and tumor (Figure 1C). Predominantly all engrafted immune cells were T cells, of which the majority were CD4^+^ T helper (Th) cells, and NOX1i did not affect CD4/CD8 T cell ratios (Figure 1D + Supplementary Figures S3A, S3B). Within the population of Th cells, mainly Th2 were detected, next to Th1, Th17 and regulatory T cells (Treg). While the donor T cell population consisted of effector and naive cells, engrafted cells mainly showed markers of effector memory cells in both Th and cytotoxic T cells, irrespective of the treatment group (Supplementary Figures S3B, S4). No differences in whole body weight during the experiment or at endpoint, and relative liver and spleen weight at endpoint, were observed (Figure 1E).

**FIGURE 2:**
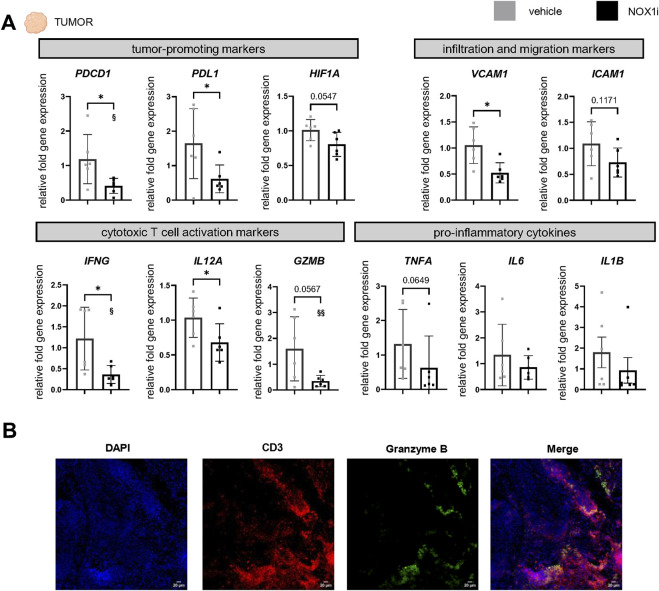
NOX1i reduces pro-tumorigenic gene expression in tumors of T cell-HIS-HCC mice. **(A)** Tumor mRNA levels of indicated markers, normalized to vehicle. Data are shown as mean (SD). P-values were calculated using unpaired t-test with Welch’s correction or unpaired Mann-Whitney U test based on normal distribution, and F-test to compare variances in cases of normal distribution. For p-values of t-test: *p < 0.05. For p-values of F-test: §p < 0.05. NOX1i: NOX1 inhibition. **(B)** Representative immunofluorescent images of CD3, representing T cells, and granzyme B in tumor tissue of T cell-HIS-HCC mice. Scale bars: 20 µm.

Hepatocarcinogenesis was confirmed on histology (Supplementary Figure S5A). NOX1i did not affect tumor growth based on tumor size, weight nor gene expression of tumor (*glypican 3* (*GPC3*)) and proliferation (*MKI67* and *PCNA*) markers (Figure 1F). However, gene expression of tumor-promoting markers, including immune checkpoints (*PDCD1* (PD1) and *PDL1*) and *HIF1A*, infiltration and migration markers (*VCAM1* and *ICAM1*), and cytotoxic T cell activation markers (*IFNG* and *IL12A*) was significantly lower in tumor tissue of NOX1i-treated mice. A similar, however non-significant, trend was observed for gene expression of pro-inflammatory cytokines (*IL6*, *IL1B* and *TNFA*) and *GZMB* (Figure 2A). NOX1i additionally reduced variability in gene expression of tumor-promoting markers (*PDCD1*) and cytotoxic T cell activation markers (*IFNG, GZMB*; Figure 2A), with GZMB protein expression co-localizing with T cells in tumor tissue of T cell-HIS-HCC mice (Figure 2B). To account for variation in humanization, we additionally normalized the gene expression to the number of engrafted human immune cells in each tumor. Upon normalization for humanization, gene expression of *GZMB* was significantly reduced following NOX1i, whereas expression of the other cytotoxic T cell activation markers and tumor-promoting, pro-inflammatory, and infiltration and migration markers showed a trend toward lower levels without reaching statistical significance. This was accompanied by significantly lower variance in gene expression in tumors of NOX1i-treated animals compared to control (Supplementary Figure S5B).

### 3.2 NOX1i reduces infiltration of human immune cells in Myeloid-HIS-HCC mice

**FIGURE 3:**
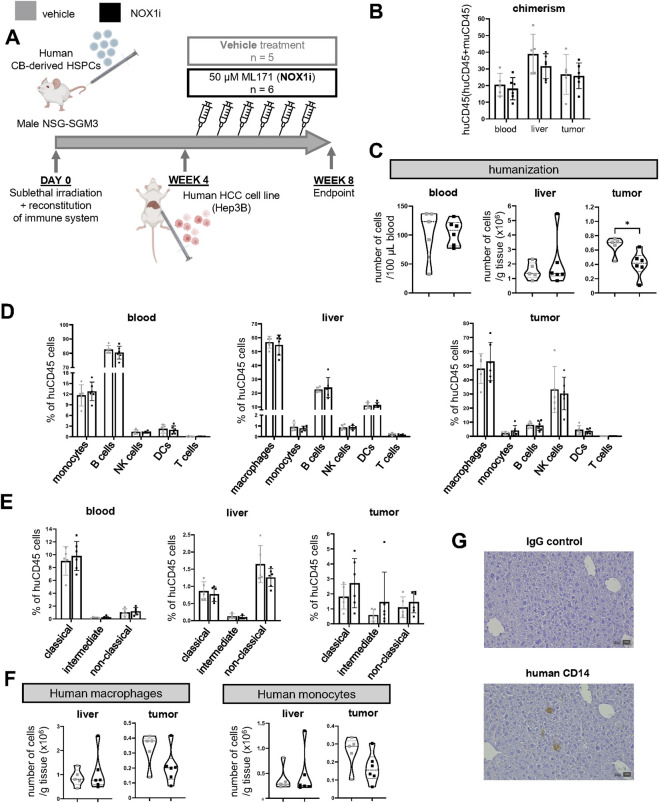
NOX1i in Myeloid-HIS-HCC mice reduces monocyte and tumor-associated macrophage infiltration. **(A)** Schematic overview of the experimental set-up. Male NSG-SGM3 mice (n = 11) were sublethally irradiated and the immune system was partly reconstituted using human cord blood (CB)-derived hematopoietic stem and progenitor cells (HSPCs). Four weeks post humanization, orthotopic human HCC was induced and mice were treated with 50 µM ML171 (NOX1i; n = 6) or vehicle (n = 5), twice per week for 3 weeks. **(B)** Human-to-murine immune cell (CD45) chimerism in the indicated tissues. **(C)** Human engraftment based on the number of human CD45 cells. Data are represented as violin plots showing median and quartiles. **(D,E)** Human immune cell subsets **(D)** and monocyte subsets **(E)** in indicated tissues represented as percentage of human CD45 cells. **(F)** Number of human macrophages and monocytes in the indicated tissues. **(G)** Representative immunohistochemistry images of infiltrated human monocytes/macrophages in liver tissue of HCC-bearing humanized mice. Sections were stained for human CD14 (brown), or matched IgG control. Scale bars: 100 µm. **(B,D,E)**. Data are shown as mean (SD). **(C–E)** Adjusted p-values were calculated using multiple unpaired t-test with Holm-Šídák correction **(D,E)**, or p-values were calculated using unpaired t-test with Welch’s correction or unpaired Mann-Whitney U test based on normal distribution **(C,E)**. *p < 0.05. HCC: hepatocellular carcinoma, NOX1i: NOX1 inhibition, huCD45: human CD45, muCD45: murine CD45, NK = natural killer, DCs: dendritic cells.

Reports show that NOX1i affects innate immunity, however, human-relevant *in vivo* evaluation in a model that interrogates innate immunity in the context of HCC is lacking. To evaluate NOX1i in the context of human HCC and human innate immune responses, NSG-SGM3 mice were sublethally irradiated and reconstituted with CB-derived HSPCs. Orthotopic human HCC was induced 4 weeks post humanization, and mice were treated with NOX1i or vehicle twice per week, for 3 weeks (Figure 3A).

In vehicle-treated animals, humanization reached 20.5%, 38.9% and 26.7% in peripheral blood, liver and tumor, respectively (Figure 3B). The infiltration of human immune cells (CD45^+^) was significantly lower in tumors of NOX1i-treated mice, compared to control, and was unaffected in blood and liver (Figure 3C). Human immune cell engraftment in peripheral blood of vehicle-treated mice mainly consisted of B cells (82.6%), whereas human immune cell engraftment was dominated by macrophages in liver (56.8%) and tumor (47.9%), with a lower proportion of B cells (22.5% in liver and 8% in tumor). Interestingly, a substantial proportion of human NK cells was found in tumor tissue (33.1%), while human NK cells were almost absent in liver (0.9%) and peripheral blood (1.4%). In addition, human dendritic cells (DCs) and monocytes were detected in peripheral blood (DCs: 2.2%; monocytes: 0.5%), liver (DCs: 11.1%; monocytes: 0.9%) and tumor (DCs: 4.6%; monocytes: 2.2%), and no human T cells were reconstituted in all analyzed organs (Figure 3D; Supplementary Figures S6, S7A). Within the monocyte population, classical (CD14^+^CD16^−^) monocytes were most prevalent in peripheral blood, next to non-classical (CD14^dim^CD16+) and intermediate (CD14^+^CD16^+^) monocytes. In the liver, human non-classical monocytes were more prevalent than classical and intermediate monocytes, while in tumor tissue all three subsets were equally represented (Figure 3E). NOX1i did not significantly affect engraftment rates of human immune cell and monocyte subsets (Figures 3D,E). However, the reduced infiltration of total human immune cells in tumors of NOX1i-treated mice was reflected by a trend toward lower infiltration of monocytes and macrophages in the tumor of NOX1i treated mice, compared to control (Figure 3F). The presence of human monocytes and macrophages in liver and tumor was validated by human CD14 immunopositivity in NOX1i-treated and control mice (Figure 3G + Supplementary Figure S7B).

**FIGURE 4:**
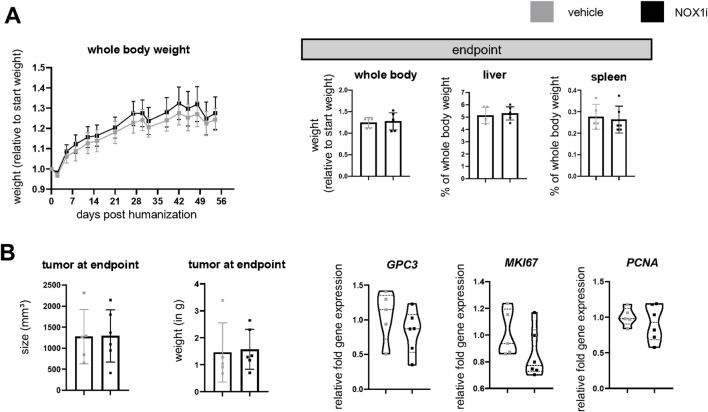
NOX1i does not affect tumor growth in Myeloid-HIS-HCC mice. **(A)** Whole body weight during the experiment and at endpoint, relative to start weight, and liver and spleen weight represented as percentage of whole body weight at endpoint. **(B)** Tumor size, weight and gene expression of *glypican 3* (*GPC3*), *Ki67* (*MKI67*) and *PCNA*. **(A,B)** Data are shown as mean (SD). P-values were calculated using unpaired t-test with Welch’s correction or unpaired Mann-Whitney U test based on normal distribution. NOX1i: NOX1 inhibition.

NOX1i did not affect whole body weight during the experiment, or relative whole body, liver and spleen weight at endpoint (Figure 4A). Tumor formation in Myeloid-HIS-HCC mice was microscopically validated (Supplementary Figure S7C). Although NOX1i did not affect macroscopic tumor growth, gene expression of tumor and proliferation markers tended to be lower in tumors of NOX1i-treated mice (Figure 4B).

**FIGURE 5:**
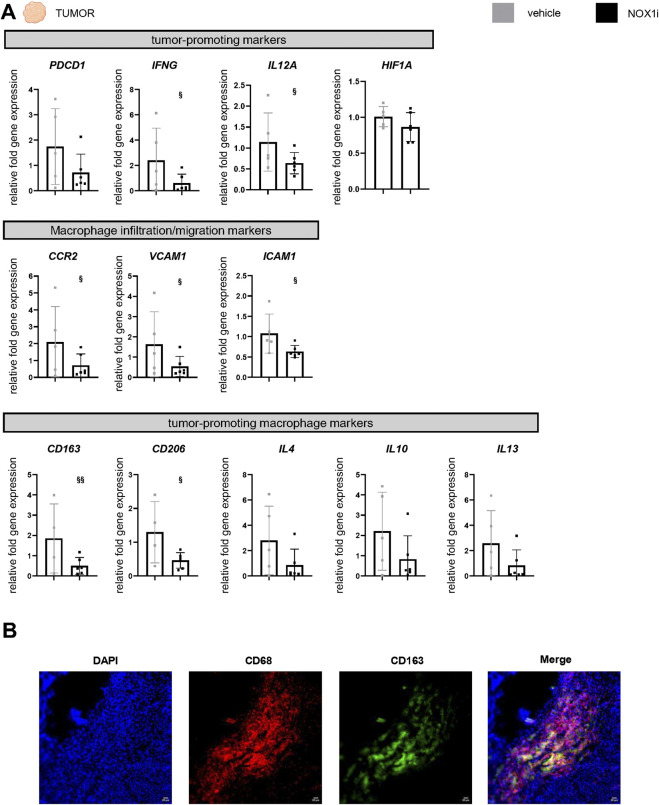
NOX1i tends to reduce tumor-promoting inflammation and M2 polarization in tumors of Myeloid-HIS-HCC mice. **(A)** Tumor mRNA levels of indicated markers. Data are shown as mean (SD). P-values were calculated using unpaired t-test with Welch’s correction or unpaired Mann-Whitney U test based on normal distribution, and F-test to compare variances in cases of normal distribution. For p-values of t-test: *p < 0.05. For p-values of F-test: §p < 0.05, §§p < 0.01. NOX1i: NOX1 inhibition. **(B)** Representative immunofluorescent images of CD68, representing macrophages, and CD163 in tumor tissue of Myeloid-HIS-HCC mice. Scale bar: 20 µm.

To further characterize the immune profile, we evaluated markers known to be associated with tumor progression and tumor-promoting macrophages. In line with the T cell-HIS-HCC model, tumors of NOX1i-treated mice showed a trend toward decreased gene expression of immune checkpoint PD1 (*PDCD1*) and inflammatory cytokines (*IFNG* and *IL12A*), of which the latter show significantly lower variability in expression upon NOX1i treatment. Gene expression of markers associated with tumor-promoting macrophages (*IL4, IL10, IL13*) followed a similar pattern. Expression of tumor-promoting macrophage markers (*CD163* and *CD206*) and leucocyte and monocyte infiltration and migration markers (*CCR2, VCAM1* and *ICAM1*) tended to be lower in tumors of NOX1i-treated mice compared to control, accompanied by significantly reduced gene expression variability (Figure 5A). Normalization of expression levels for humanization did not majorly affect the results (Supplementary Figure S8). In addition, protein expression of tumor-promoting macrophage marker CD163 co-localizes with macrophages, identified by CD68 immunofluorescent staining (Figure 5B).

### 3.3 NOX1i reduces the pro-tumorigenic environment in patient-derived precision-cut tumor slices

**FIGURE 6:**
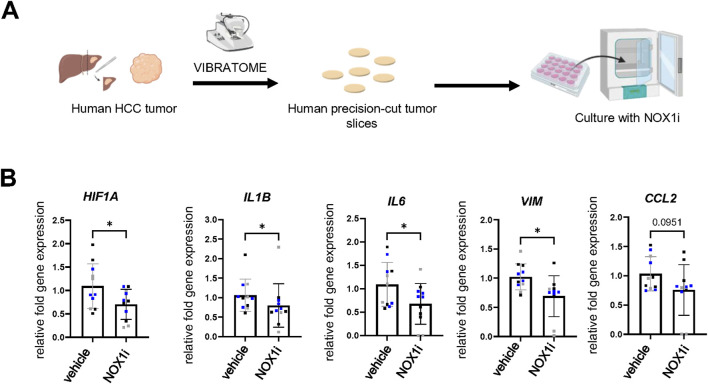
NOX1i reduces the pro-tumorigenic environment in patient-derived precision-cut tumor slices. **(A)** Overview of the experimental set-up. PCTS (n = 22) of 3 donors were cultured with NOX1i (n = 11) or vehicle (n = 11) for 6 h. **(B)** Gene expression of the indicated markers. One point indicates one slice (n = 22), one color indicates one donor (n = 3). Data are shown as mean (SD). P-values are calculated using unpaired t-test with Welch’s correction or unpaired Mann-Whitney U test based on normal distribution. *p < 0.05. HCC: hepatocellular carcinoma, NOX1i: NOX1 inhibition, PCTS: precision-cut tumor slices.

To increase translation of our results to the clinic, *ex vivo* patient-derived precision-cut tumor slices were generated from three HCC patients and cultured with NOX1i (Figure 6A). *Ex vivo* treatment of patient-derived PCTS with NOX1i showed a significantly decreased gene expression of tumor-promoting markers, including *HIF1A*, *IL6*, *IL1B* and *VIM*, and a trend toward decreased expression for *CCL2*, in line with results obtained in our HIS models (Figure 6B). To assess donor variability, we performed one-way ANOVA for each gene and condition. No significant donor differences were observed in the vehicle condition. Upon NOX1i, *CCL2* (p = 0.02) and *IL1B* (p = 0.046) significantly differed between two of three donors. *HIF1A*, *IL6*, and *VIM* remained consistent across donors in both conditions.

## 4 Discussion

In our study, we show that NOX1i modulates the TIME in three human-relevant HCC models. Previously, NOX1i has shown to alter the TME of murine HCC (Liang et al., 2019; Vandierendonck et al., 2021; Mu et al., 2025). Considering the substantial differences between the murine and human TME, including immune cells, we developed two novel humanized immune system mouse models that interrogate human HCC in the context of adaptive immunity (T cell-HIS-HCC) or innate immunity (Myeloid-HIS-HCC). In particular, T cell-HIS-HCC mice predominantly engraft human T cells, whereas Myeloid-HIS-HCC mice demonstrate high reconstitution of human macrophages in liver and tumor tissue, which has not yet been achieved for liver disease (Weinfurtner et al., 2025; Zhao et al., 2021). To increase clinical translation, we leveraged *ex vivo* patient-derived PCTS, complementary to the *in vivo* models. All three human-relevant models allow investigation of human HCC in the context of human immunity (innate immunity, adaptive immunity or tumor-infiltrated immune cells) and enhance clinical relevance and translation.

Our work shows a trend of decreased infiltration of human immune cells in NOX1i-treated T cell-HIS-HCC mice, accompanied by a decreased gene expression of immune cell infiltration markers (*VCAM1, ICAM1*), compared to control mice. Likewise, infiltration of immune cells in the Myeloid-HIS-HCC model was decreased in tumors of NOX1i-treated mice. As in the T cell-HIS-HCC model, tumors of NOX1i-treated Myeloid-HIS-HCC mice show a trend toward decreased gene expression of macrophage and leucocyte infiltration and migration markers (*CCR2*, *ICAM1* and *VCAM1).* Moreover, *ex vivo* PCTS treated with NOX1i show a trend toward decreased gene expression of *CCL2*, known to attract monocytes to the tumor (Donne and Lujambio, 2023). This aligns with previous research that links high NOX1-derived ROS levels with increased VCAM1 and ICAM1 expression and TAM recruitment (Cheung and Vousden, 2022; Yu et al., 2024), and is in line with our previous research on NOX1i in an immunocompetent murine HCC model (Vandierendonck et al., 2021). Given the tumor-promoting capacity of TAMs, decreasing the infiltration in the tumor has been proposed as therapeutic strategy for solid cancers, including HCC (Li et al., 2017), and suggests a favorable effect of NOX1i as TIME-modulating therapeutic strategy for human HCC.

NOX1-derived ROS regulate VCAM1 and ICAM1 expression via NF-kB and NRF2 pathways (Shah et al., 2024). Notably, these pathways additionally regulate HIF1A expression (Taylor and Scholz, 2022) as evident by the increased HIF-1α gene and protein expression by NOX1-produced ROS in cancer cells *in vitro* (Cheung and Vousden, 2022; Goyal et al., 2004). Consistent with this, our data show decreased gene expression of *HIF1A* in NOX1i-treated PCTS, and a trend toward decreased *HIF1A* levels in tumor tissue of both NOX1i-treated HIS-HCC mouse models.

In addition, ROS affects NF-kB and NRF2 pathways to regulate pro-inflammatory cytokine expression, including IL-6, IL-1β and TNF-α, suggesting that reduction of ROS through NOX1i might result in decreased levels of these markers. Indeed, NOX1i reduces *il1b* and *il6* levels in an immunocompetent murine HCC model (Mu et al., 2025; Vandierendonck et al., 2021). More importantly, NOX1i in our human-relevant models also reduced pro-inflammatory markers as demonstrated by a significantly decreased gene expression of *IL6* and *IL1B* in NOX1-treated PCTS, and a trend toward lower gene expression levels of *IL1B*, *IL6* and *TNFA* in tumor tissue of both HIS-HCC models.

High ROS levels contribute to uncontrolled inflammation and increased expression of immune checkpoints in macrophages, and drive polarization toward the M2 phenotype (Shah et al., 2024; Yu et al., 2024; Roux et al., 2019; Glorieux et al., 2024). This hypothesis is supported by the immune profile in our Myeloid-HIS-HCC mouse model, which showed a trend toward decreased immune checkpoints and tumor-promoting, immunosuppressive cytokines and markers associated with M2 phenotype and TAMs (*IL4, IL10, IL13, CD163, CD206, IFNG, IL12A*) in tumor tissue of NOX1i-treated mice. Despite IFNG is considered as a marker of anti-tumor immunity, chronic IFNG expression supports tumor growth and results in T cell exhaustion, especially in combination with IL-10. Interestingly, this mechanism is hijacked by tumor cells to suppress immunity (Shalapour and Karin, 2019) and the lower expression of *IFNG* in both our HIS-HCC models, and additionally *IL10* in the Myeloid-HIS-HCC model, further support the potential of NOX1i to reshape the TIME and break the tumor-permissive environment established by tumor cells. Considering that gene expression was evaluated in whole tumor tissue rather than in separate tissue components, the reduced expression of tumor-promoting markers in both HIS-HCC models may reflect the trend toward decreased human immune cell infiltration in tumor tissue of both models. Therefore, we also analyzed gene expression normalized to the number of engrafted human immune cells. Importantly, the observed trends partially persisted after normalization, indicating that the reduced gene expression in tumors of NOX1i cannot be explained solely by lower immune cell infiltration. This suggests that NOX1i not only affects immune cell infiltration, but also modulates tumor and/or immune cell function. The latter is strengthened by the co-localization of T cells or macrophages with markers that were altered upon NOX1i in the T cell-HIS-HCC or Myeloid-HIS-HCC model, respectively.

Cytotoxic T cell function is a major effector mechanism of anti-tumor immunity and is largely mediated through both direct cytotoxic mechanisms (e.g., granzyme B) and cytokine production (e.g., IFN-γ) (Chi et al., 2024). In tumor tissue of NOX1i-treated T cell-HIS-HCC mice, the trend toward reduced *GZMB* expression and significant decrease in *IFNG* and *IL12A* gene expression, the significant reduction in *GZMB* expression after normalization for human immune cell infiltration, and the co-localization of granzyme B with T cells in tumor tissue of NOX1i-treated mice collectively suggest that NOX1i may impair human T cell function in our model. However, alongside reduced cytotoxic T cell markers, tumor-promoting markers were also decreased, indicative of an overall attenuation of inflammation in the TIME of NOX1i-treated T cell-HIS-HCC mice. These findings further emphasize the value of employing two complementary humanized models engrafted with either innate or adaptive immune cells. While the Myeloid-HIS-HCC model primarily indicates reduced tumor-promoting responses, with a similar shift observed in patient-derived PCTS, the T cell model suggests a reduction in both pro-tumor and anti-tumor effects, including inflammation. Importantly, while acute inflammation is favored for effective anti-cancer immune responses, disrupting chronic inflammation in inflammation-driven cancer, including HCC, might sensitize the tumor for other treatments or prevent resistance to conventional treatment strategies (Pelly et al., 2021; Lu et al., 2018), highlighting the potential of NOX1i as combination therapy in HCC, irrespective of potentially reduced cytotoxic T cell function. In addition, we here present three human-relevant models to evaluate cancer immunity which can be leveraged by the broader research community.

## Data Availability

The original contributions presented in the study are included in the article/Supplementary Material, further inquiries can be directed to the corresponding author.
