# Supplementary material for: Harnessing human immune system models to validate NADPH oxidase 1 inhibition as treatment for hepatocellular carcinoma
Source: Front Pharmacol. 2026 Jul 14;17:1808515. doi: 10.3389/fphar.2026.1808515 (PMC13407187; doi:10.3389/fphar.2026.1808515)
Supplement: Supplementary file 1 [file Supplementaryfile1.docx]

Supplementary Material

# Supplementary Tables

**Supplementary Table S1. Patient characteristics.** NA: not applicable

| **Patient ID** | **Age** | **Sex** | **Etiology** | **Underlying liver disease** |
| --- | --- | --- | --- | --- |
| HCC01 | 83 | Male | NA | NA |
| HCC02 | 67 | Male | Ethyl | fibrosis |
| HCC03 | 52 | Male | Hepatitis B virus | fibrosis |

**Supplementary Table S2. Flow cytometry panels**

**Hematopoietic stem and progenitor cell purity panel**

| **Antigen** | **Clone** | **Fluorophore** | **Species** | **Catalog number** | **dilution** |
| --- | --- | --- | --- | --- | --- |
| CD3 | SK7 | AF488 | Human | 344810 | 1/200 |
| CD34 | 581 | PE | Human | 343506 | 1/100 |

**Human immune cell chimerism panel**

| **Antigen** | **Clone** | **Fluorophore** | **Species** | **Catalog number** | **dilution** |
| --- | --- | --- | --- | --- | --- |
| CD45 | HI30 | APC-fire750 | Human | 304062 | 1/200 |
| CD45 | 30-F11 | AF700 | Murine | 103128 | 1/200 |

**Human monocyte/macrophage panel**

| **Antigen** | **Clone** | **Fluorophore** | **Species** | **Catalog number** | **dilution** |
| --- | --- | --- | --- | --- | --- |
| CD3 | SK7 | AF488 | Human | 344810 | 1/200 |
| CD14 | M5E2 | BV785 | Human | 301840 | 1/50 |
| CD16 | 3G8 | PE-Cy7 | Human | 302015 | 1/1600 |
| CD19 | HIB19 | PerCP-Cy5.5 | Human | 302230 | 1/50 |
| CD45 | HI30 | APC-fire750 | Human | 304062 | 1/200 |
| CD45 | 30-F11 | AF700 | Murine | 103128 | 1/200 |
| CD56 | HCD56 | BV421 | Human | 318328 | 1/50 |
| HLA-DR | L243 | BV605 | Human | 307640 | 1/50 |
| CD68 | Y1/82A | PE | Human | 333808 | 1/100 |

**Human T cell-specific panel**

| **Antigen** | **Clone** | **Fluorophore** | **Species** | **Catalog number** | **dilution** |
| --- | --- | --- | --- | --- | --- |
| CD3 | SK7 | AF488 | Human | 344810 | 1/200 |
| CD4 | RPA-T4 | PerCP-Cy5.5 | Human | 300530 | 1/50 |
| CD8 | SK1 | AF700 | Human | 344724 | 1/200 |
| CD45RA | HI100 | BV785 | Human | 304140 | 1/100 |
| CD45 | HI30 | APC-fire750 | Human | 304062 | 1/200 |
| CD45 | 30-F11 | BV570 | Murine | 103135 | 1/100 |
| CXCR3 (CD183) | G025H7 | PE-Dazzle594 | Human | 353736 | 1/100 |
| CCR6 | G034E3 | PE-Cy7 | Human | 353418 | 1/400 |
| CCR7 (CD197) | G043H7 | PE | Human | 353204 | 1/50 |
| HLA-DR | L243 | BV605 | Human | 307640 | 1/50 |
| CD25 | M-A251 | BV711 | Human | 356138 | 1/25 |
| FOXP3 | 259D | AF647 | Human | 320214 | 1/100 |

**Supplementary Table S3. Human primer sequences**

| **Human reference gene** | **Forward primer** | **Reverse primer** |
| --- | --- | --- |
| HPRT | 5’-TGACACTGGCAAAACAATGCA-3’ | 5’-GGTCCTTTCACCAGCAAGCT-3’ |
| HMBS | 5’-GGCAATGCGGCTGCAA-3’ | 5’-GGGTACCCACGGAATCAC-3’ |
| ACTB | 5’-GGACTTCGAGCAAGAGATGG-3’ | 5’-AGCACTGTGTTGGCGTACAG-3’ |
| SDHA | 5-TGGGAACAAGAGGGCATCTG-3’ | 5’-CCACCACTGCATCAAATTCATG-3’ |
|  |  |  |
| **Human target gene** | **Forward primer** | **Reverse primer** |
| GPC3 | 5’-CCTTTGAAATTGTTGTTCGCCA-3’ | 5’-CCTGGGTTCATTAGCTGGGTA-3’ |
| MKI67 | 5’-GCCTGCTCGACCCTACAGA-3’ | 5’-GCTTGTCAACTGCGGTTGC-3’ |
| PCNA | 5’-GCGTGAACCTCACCAGTATGT-3’ | 5’-TCTTCGGCCCTTAGTGTAATGAT-3’ |
| PDCD1 | 5’-GCCTGTGTTCTCTGTGGACT-3’ | 5’-CATACTCCGTCTGCTCAGGG-3’ |
| PDL1 | 5’-TGCCGACTACAAGCGAATTACTG-3’ | 5’-CTGCTTGTCCAGATGACTTCGG-3’ |
| HIF1A | 5’-TGCCAGCTCAAAAGAAAACA-3’ | 5’-ACCAACAGGGTAGGCAGAAC-3’ |
| VCAM1 | 5’-CCTGGACCCCGGATTGCTGC-3’ | 5’-ACCTTCCCGCTCAGAGGGCT-3’ |
| ICAM1 | 5’-ACGGATGCCAGCTTGGGCAC-3’ | 5’-GGGAGCTCCGTGAGGCCAGA-3’ |
| IFNG | 5’-ACTGACTTGAATGTCCAACGCA-3’ | 5’-ATCTGACTCCTTTTTCGCTTCC-3’ |
| IL12A | 5’-CCACAAAAATCCTCCCTTGA-3’ | 5’-AAATGACAACGGTTTGGAGG-3’ |
| TNF | 5’-CCTGCCCCAATCCCTTTATT-3’ | 5’-CCCTAAGCCCCCAATTCTCT-3’ |
| IL6 | 5’-GGCACTGGCAGAAAACAACC-3’ | 5’-GCAAGTCTCCTCATTGAATCC-3’ |
| IL1B | 5’-CACGATGCACCTGTACGATCA-3’ | 5’-GTTGCTCCATATCCTGTCCCT-3’ |
| CD163 | 5’-GCGGGAGAGTGGAAGTGAAAG-3’ | 5’-GTTACAAATCACAGAGACCGCT-3’ |
| CD206 | 5’-GGGTTGCTATCACTCTCTATGC-3’ | 5’- TTTCTTGTCTGTTGCCGTAGTT-3’ |
| IL4 | 5’-TATGCAAAGCAAAAAGCCAG-3’ | 5’-TGTTCCTGTGAAATCAGACCA-3’ |
| IL10 | 5’-GACTTTAAGGGTTACCTGGGTTG-3’ | 5’-TCACATGCGCCTTGATGTCTG-3’ |
| IL13 | 5’-TCATGGCGCTTTTGTTGACC-3’ | 5’-GAGCCTTCTGGTTCTGGGTG-3’ |
| CCR2 | 5’-TGCAAAAAGCTGAAGTGCTTG-3’ | 5’- CAGCAGAGTGAGCCCACAAT-3’ |
| VIM | 5’-AGTCCACTGAGTACCGGAGAC-3’ | 5’- CATTTCACGCATCTGGCGTTC-3’ |
| CCL2 | 5’-GAAAGTCTCTGCCGCCCTT-3’ | 5’- GCATTGATTGCATCTGGCTGAG-3’ |

# Supplementary Figures


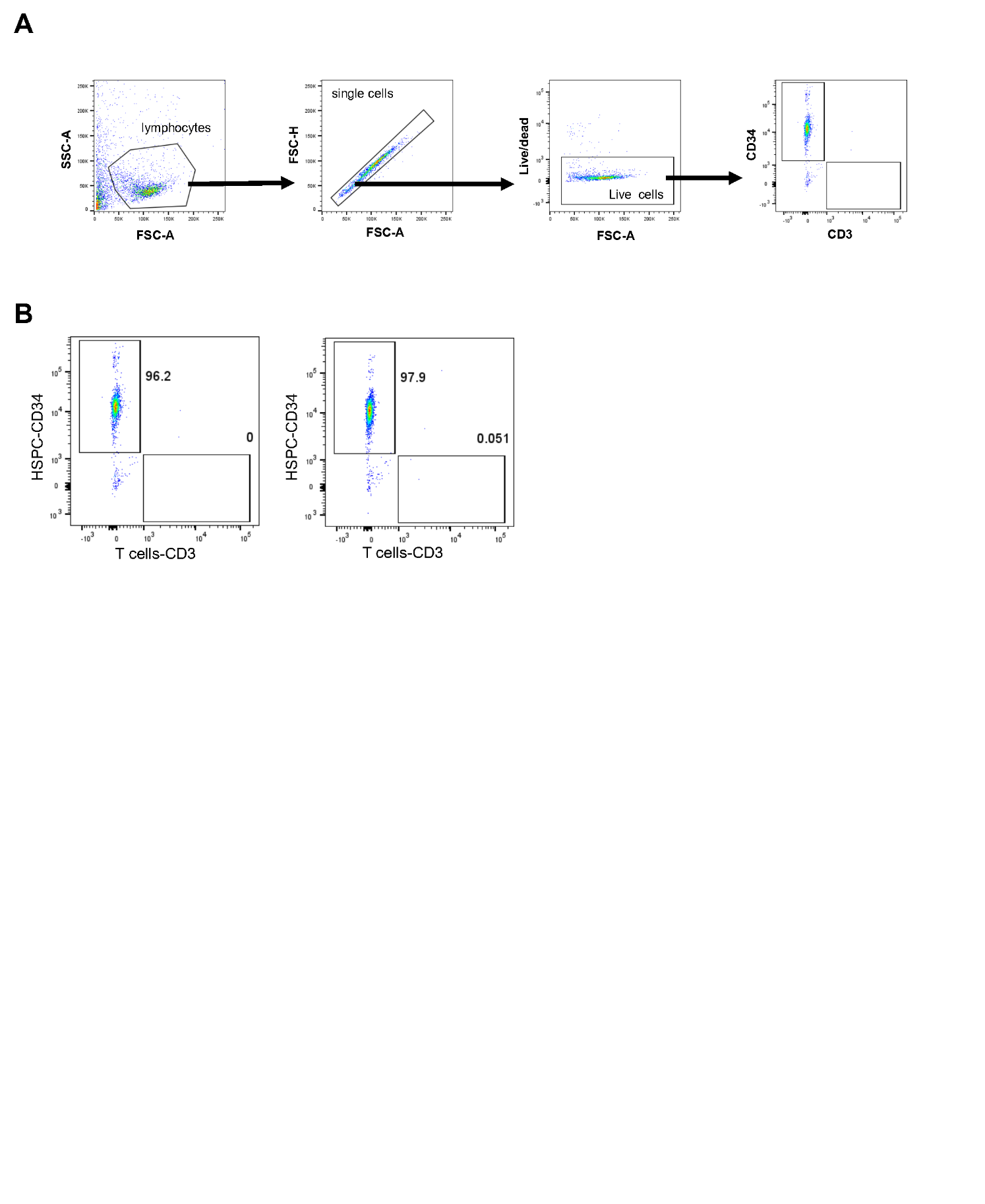


**Supplementary Figure S1.** (**A**) Representative gating strategy of the hematopoietic stem and progenitor cell purity panel. (**B**) Dot plots evaluating purity of cord blood-derived hematopoietic stem and progenitor cells (HSPCs) based on CD34+ expression and absence of T cells (CD3).


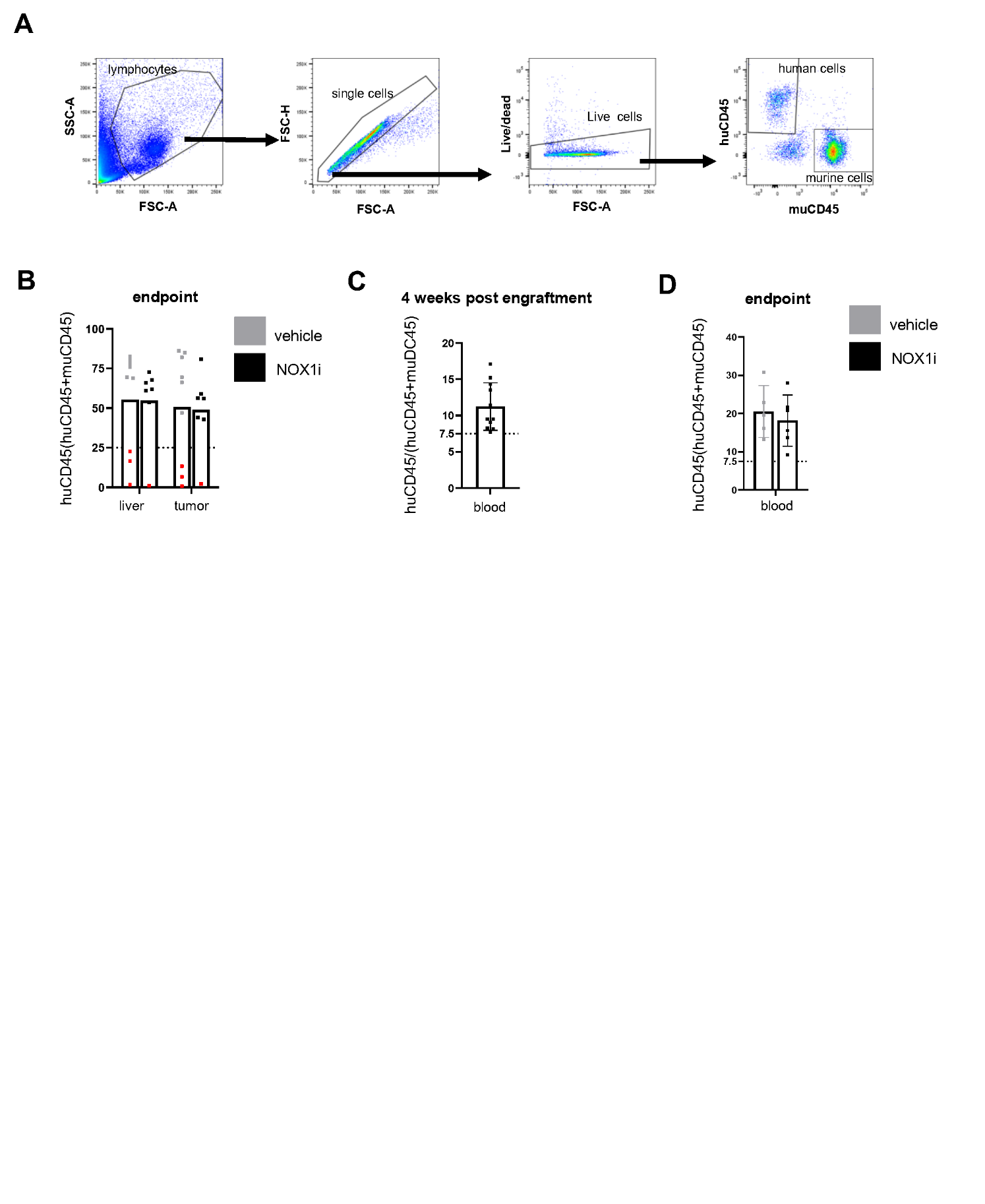


**Supplementary Figure S2.** (**A**) Representative gating strategy of the human immune cell chimerism panel. (**B**) Validation of successful engraftment of T cell-HIS-HCC mice at endpoint, represented as human-to-murine immune cell chimerism. The dotted line (25%) indicates threshold for inclusion in analyses (pre-defined criterium), and red dots indicate excluded mice. Data are shown as mean. (**C-D**) Validation of successful engraftment in peripheral blood of Myeloid-HIS-HCC mice 4 weeks post humanization, prior to HCC induction (**C**), or at endpoint (**D**). The dotted line (7.5%) indicates threshold for inclusion in the experiment (predefined criterium). Data are shown as mean (SD). HIS: humanized immune system, HCC: hepatocellular carcinoma, NOX1i: NOX1 inhibition, huCD45: human CD45, muCD45: murine CD45.


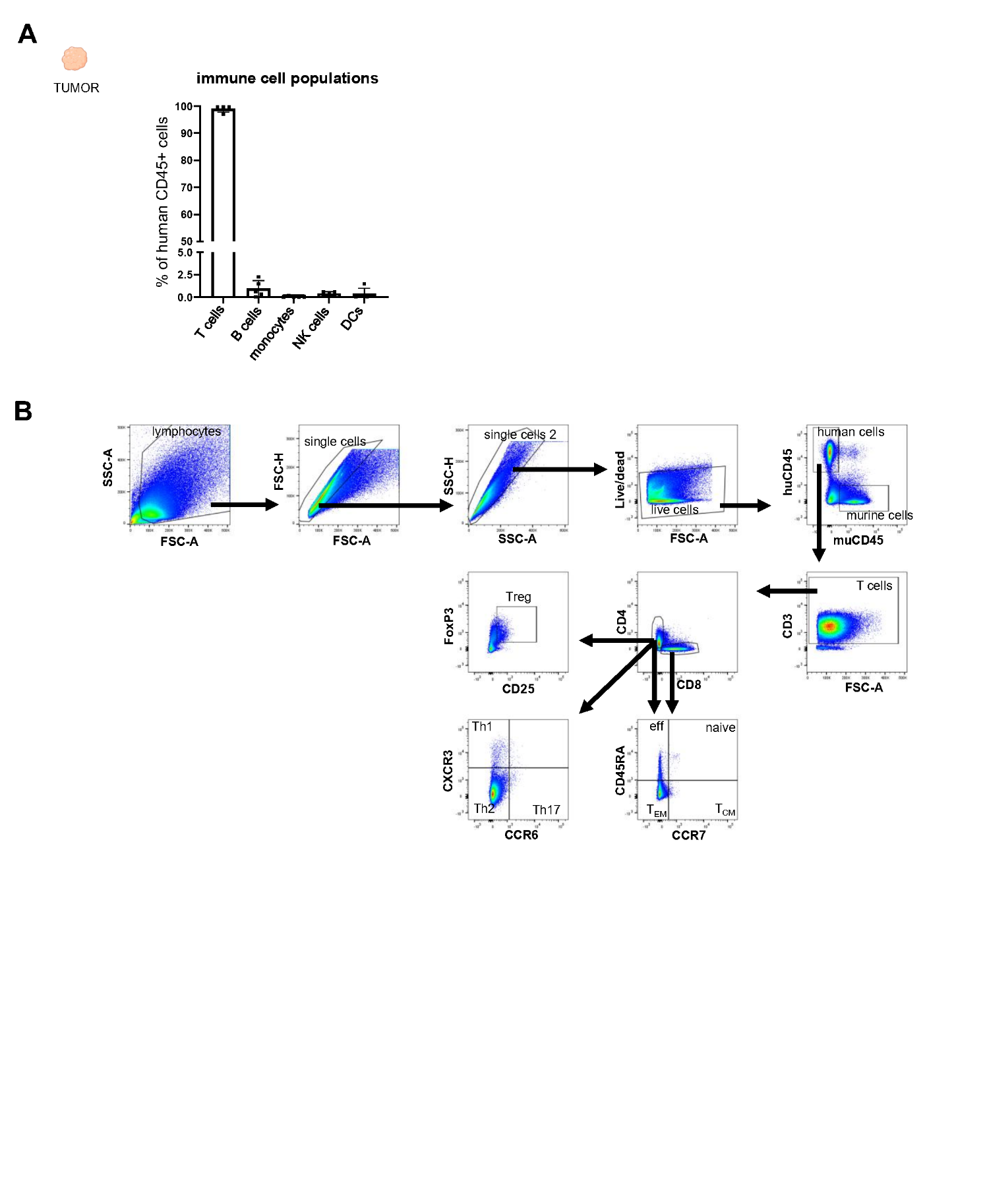


**Supplementary Figure S3.** (**A**) Representative engraftment of human immune cell populations, shown as percentage of human CD45+ cells, in tumor tissue of T cell-HIS-HCC mice at 3 weeks post engraftment. Data are shown as mean (SD). NK: natural killer, DCs: dendritic cells. (**B**) Representative gating strategy of the human T cell-specific panel. huCD45: human CD45, muCD45: murine CD45, eff: effector, T_EM_: effector memory T cell, T_CM_: central memory T cell, Treg: regulatory T cell, Th: helper T cell.

**
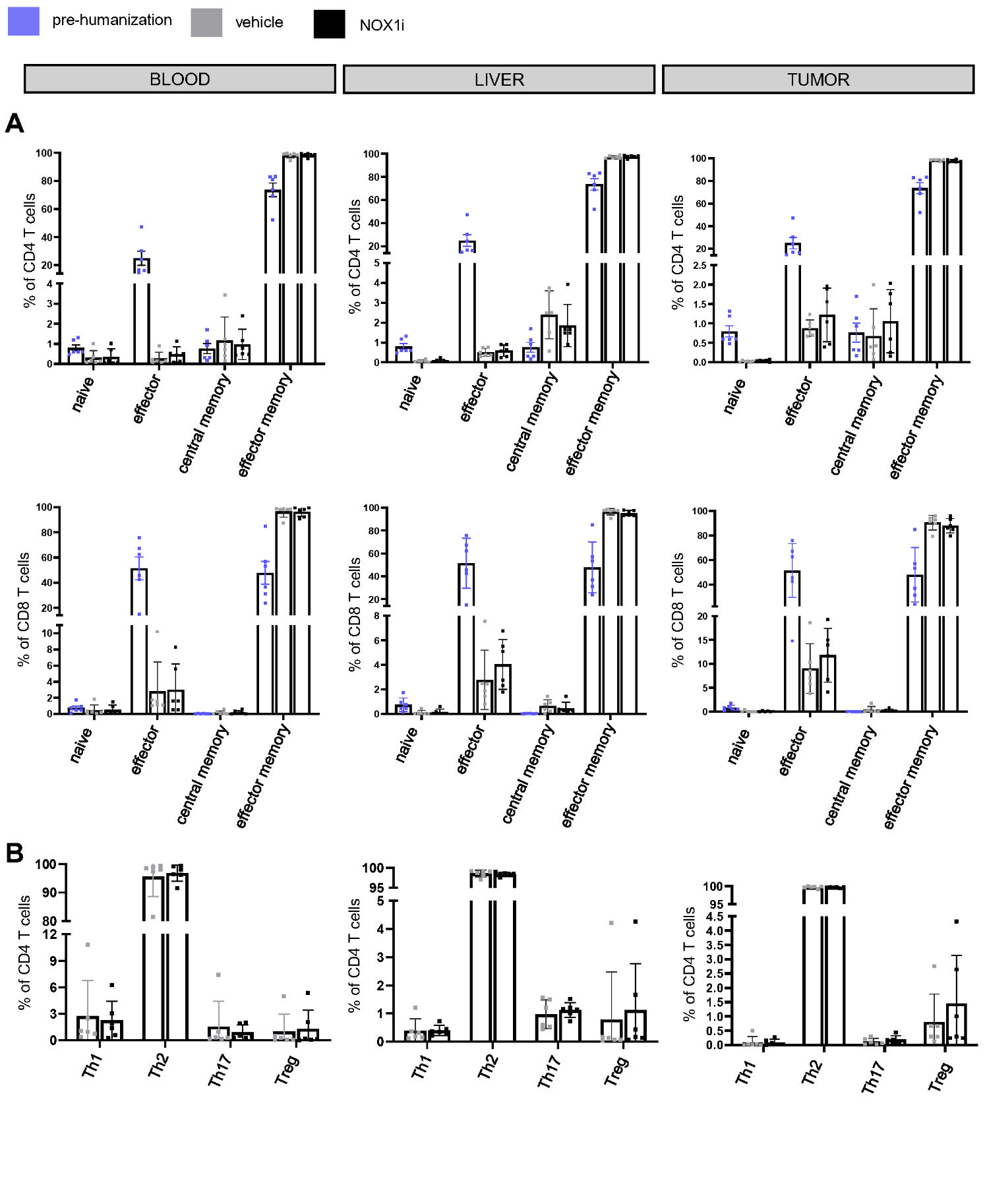
Supplementary Figure S4**. (**A**) CD4 (Thelper, Th) and CD8 T cell differentiation states as percentage of CD4 or CD8 T cells in the injected fraction (pre-humanization), or peripheral blood, liver and tumor at endpoint. (**B**) Th subsets (Th1, Th2, Th17, regulatory T cell (Treg)) at endpoint in the indicated tissues, shown as percentage of CD4 (Th) cells. (**A-B**) Data are shown as mean (SD). P-values were calculated using unpaired multiple t-test with Welch’s correction and multiple comparisons.


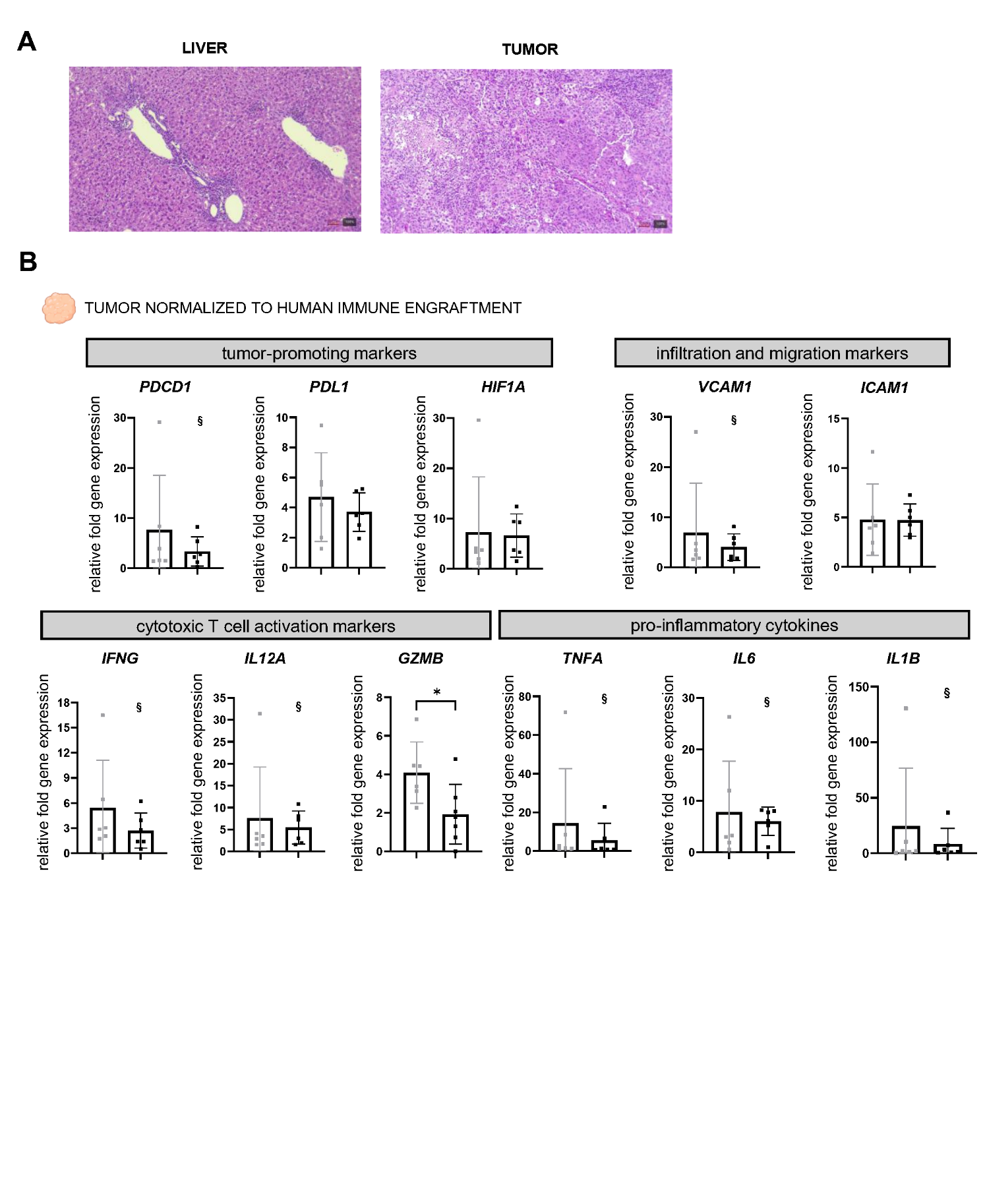


**Supplementary Figure S5**. (**A**) Representative images of hematoxylin and eosin staining in liver and tumor tissue of T cell-HIS-HCC mice. Scale bar = 100 µm. (**B**) Tumor mRNA levels of indicated markers normalized to the number of engrafted human immune cells in tumor tissue of T cell-HIS-HCC mice. Data are shown as mean (SD). P-values were calculated using unpaired t-test with Welch’s correction or unpaired Mann-Whitney U test based on normal distribution, and F-test to compare variances in cases of normal distribution. For p-values of t-test: * p < 0.05. For p-values of F-test: § p < 0.05.

**
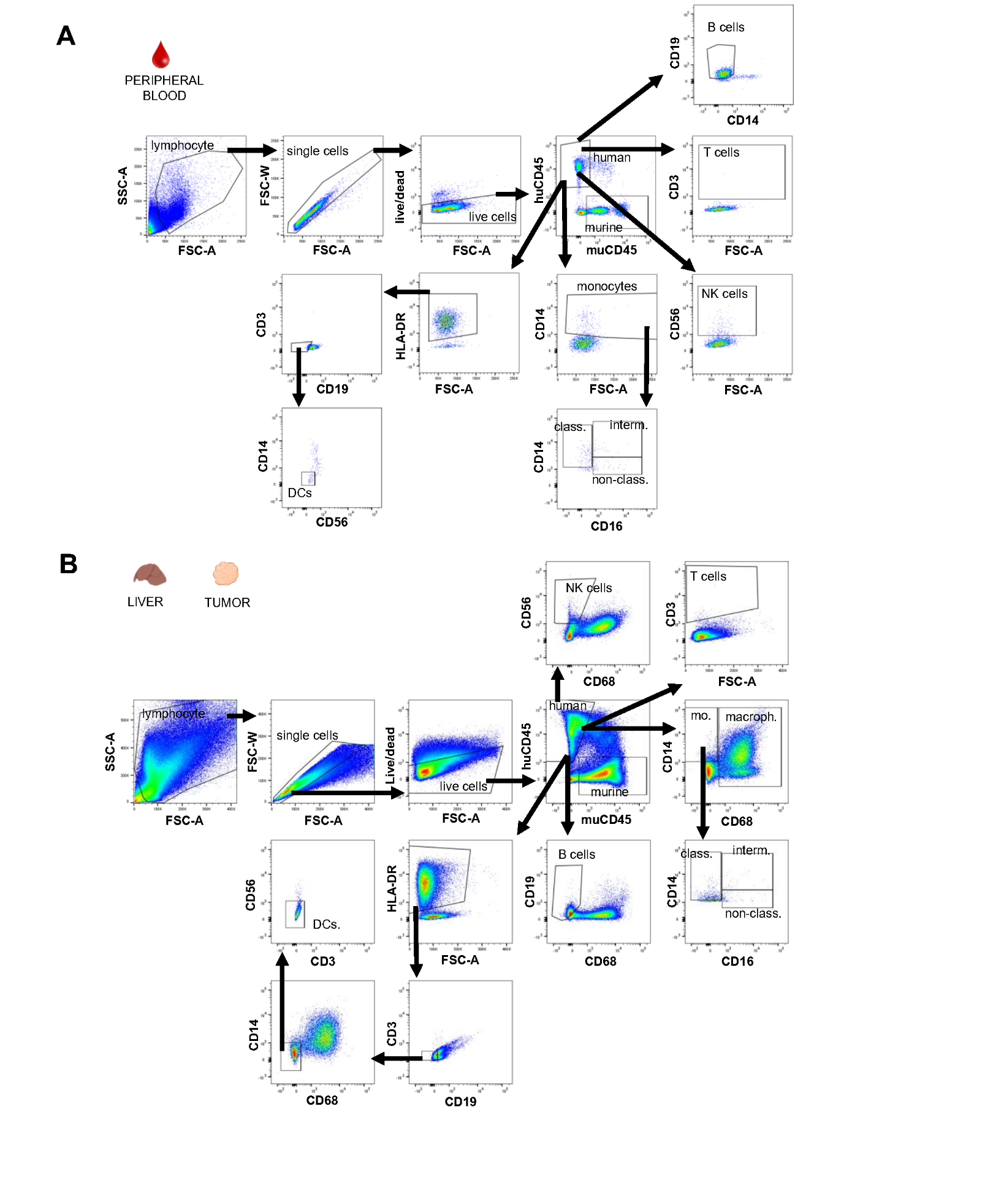
Supplementary Figure S6.** Representative gating strategy of the monocyte/macrophage panel in peripheral blood (**A**) and liver and tumor (**B**). huCD45: human CD45, muCD45: murine CD45, mo: monocyte, macroph.: macrophage, class.: classical monocyte, interm.: intermediate monocyte, non-class.: non-classical monocyte, DCs: dendritic cells, NK: natural killer.

**
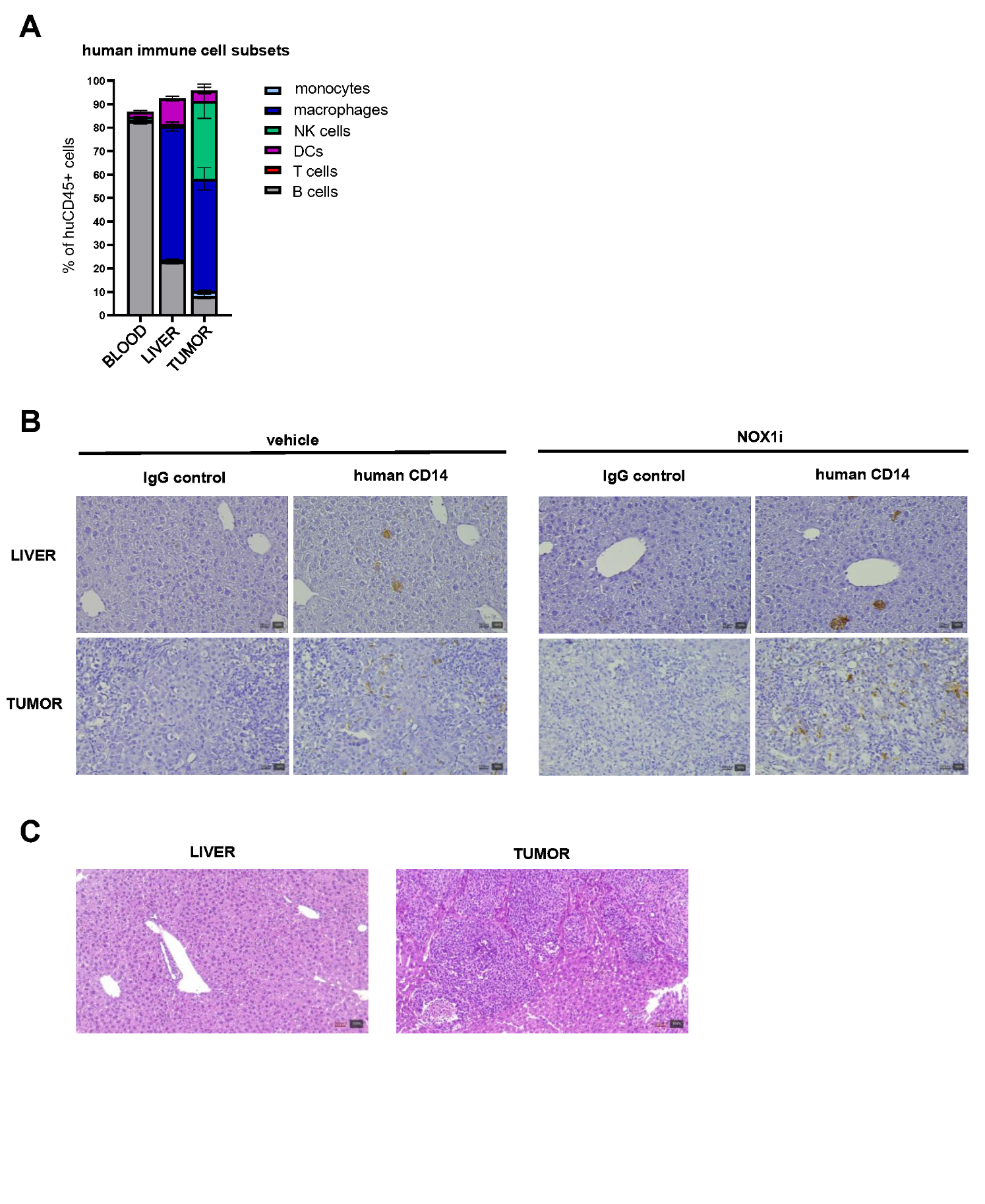
Supplementary Figure S7.** (**A**) Human immune cell subsets in indicated tissues of vehicle-treated mice. data are shown as mean (SD). NK: natural killer, DCs: dendritic cells. (**B**) Representative hematoxylin and eosin images of liver and tumor tissue of Myeloid-HIS-HCC mice. Scale bars: 100 µm. (**C**) Representative immunohistochemistry images of infiltrated human monocytes/macrophages in liver and tumor tissue of HCC-bearing humanized mice. Sections were stained for human CD14 (brown), or matched IgG control. Scale bars: 100 µm.

**
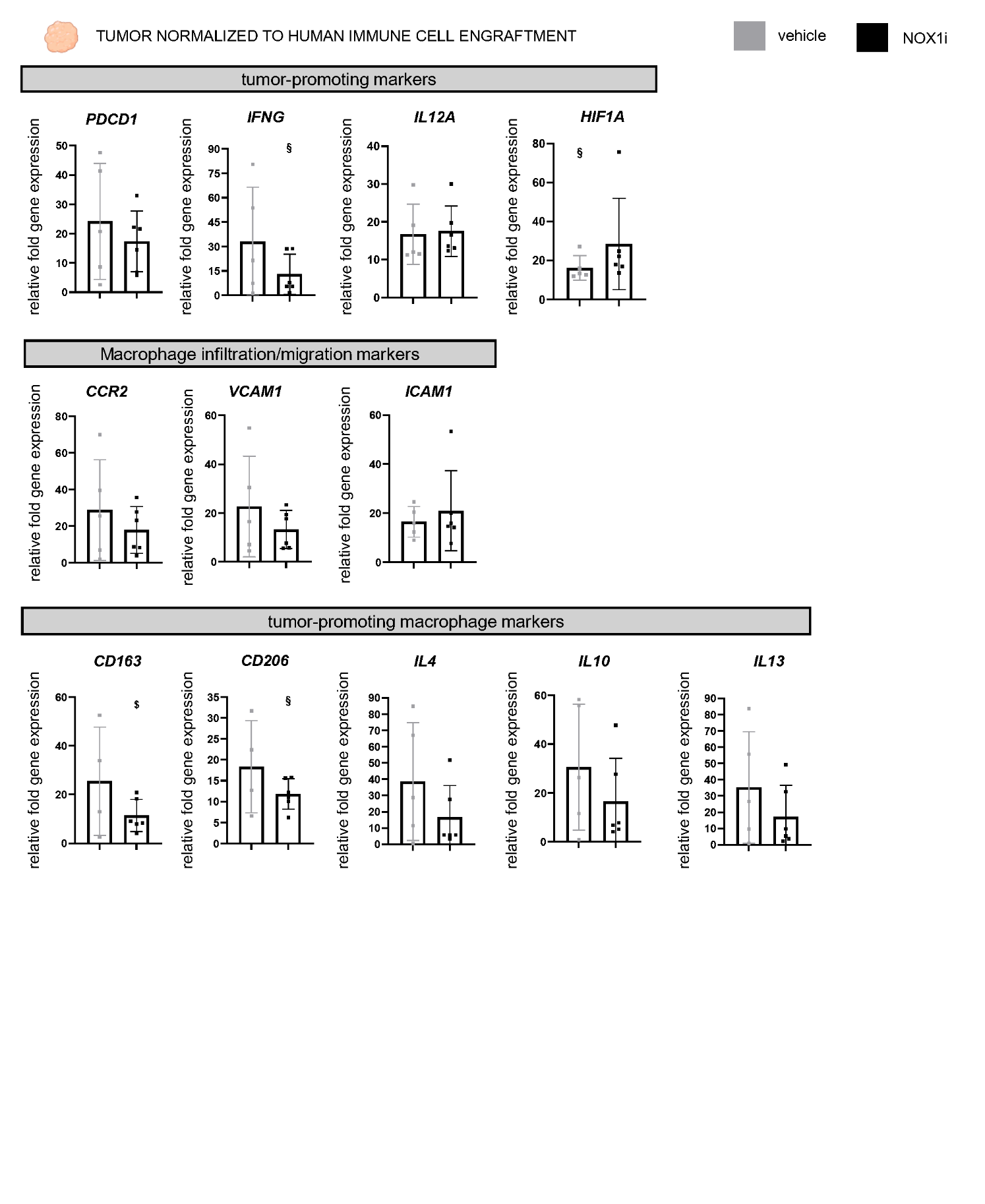
Supplementary Figure S8.** Tumor mRNA levels of indicated markers normalized to the number of engrafted human immune cells in the tumor of Myeloid-HIS-HCC mice. Data are shown as mean (SD). P-values were calculated using unpaired t-test with Welch’s correction or unpaired Mann-Whitney U test based on normal distribution, and F-test to compare variances in cases of normal distribution. For p-values of F-test: § p < 0.05.
